# Endotoxin levels correlate positively with a sedentary lifestyle and negatively with highly trained subjects

**DOI:** 10.1186/1476-511X-9-82

**Published:** 2010-08-04

**Authors:** Fabio S Lira, Jose C Rosa, Gustavo D Pimentel, Hélio A Souza, Erico C Caperuto, Luiz C Carnevali, Marília Seelaender, Ana R Damaso, Lila M Oyama, Marco T de Mello, Ronaldo V Santos

**Affiliations:** 1 Department of Physiology, Division of Nutrition Physiology, Universidade Federal de São Paulo - UNIFESP, São Paulo, SP, Brazil; 2 Cancer Metabolism Group, Institute of Biomedical Sciences, Department of Cell and Developmental Biology, University of São Paulo, Brazil; 3 Department of Physical Education, Biology and Health Sciences Center, Mackenzie Presbyterian University, São Paulo, Brazil; 4 Department of Bioscience, Baixada Santista Campus, Universidade Federal de São Paulo - UNIFESP, São Paulo, SP, Brazil; 5 Department of Psychobiology, Universidade Federal de São Paulo - UNIFESP, São Paulo, SP, Brazil

## Abstract

**Introduction:**

A sedentary lifestyle increases the risk of developing cardiovascular disease, obesity, and diabetes. This phenomenon is supported by recent studies suggesting a chronic, low-grade inflammation status. Endotoxin derived from gut flora may be key to the development of inflammation by stimulating the secretion of inflammatory factors. This study aimed to examine plasma inflammatory markers and endotoxin levels in individuals with a sedentary lifestyle and/or in highly trained subjects at rest. Methods: Fourteen male subjects (sedentary lifestyle n = 7; highly trained subjects n = 7) were recruited. Blood samples were collected after an overnight fast (~12 h). The plasmatic endotoxin, plasminogen activator inhibitor type-1 (PAI-1), monocyte chemotactic protein-1 (MCP1), ICAM/CD54, VCAM/CD106 and lipid profile levels were determined.

**Results:**

Endotoxinemia was lower in the highly trained subject group relative to the sedentary subjects (p < 0.002). In addition, we observed a positive correlation between endotoxin and PAI-1 (r = 0.85, p < 0.0001), endotoxin and total cholesterol (r = 0.65; p < 0.01), endotoxin and LDL-c (r = 0.55; p < 0.049) and endotoxin and TG levels (r = 0.90; p < 0.0001). The plasma levels of MCP-1, ICAM/CD54 and VCAM/CD106 did not differ.

**Conclusion:**

These results indicate that a lifestyle associated with high-intensity and high-volume exercise induces favorable changes in chronic low-grade inflammation markers and may reduce the risk for diseases such as obesity, diabetes and cardiovascular diseases.

## Introduction

A sedentary lifestyle increases the risk of developing cardiovascular disease and diabetes [1,2], as well as many other diseases that are linked to inflammatory markers in the plasma [3]. In previous work, we showed that sedentary subjects present a favorable state that leads to hypertriglyceridemia and hypercholesterolemia together with high levels of plasminogen activator inhibitor type-1 (PAI-1). On the other hand, the performance of highly trained athletes and a large amount of exercise significantly reduced the possibility of risk for cardiovascular diseases [4].

In recent years, it has been hypothesized that the microbial ecology in humans could be an important factor in energy homeostasis (i.e., obesity, diabetes, fatty liver) [5-10]. Lipopolysaccharide (LPS), which is also referred to as endotoxin, has been implicated as potentially important in this respect - as it is a potent inducer of inflammation. Under normal circumstances, only small amounts of endotoxin will cross from the intestinal lumen into the systemic circulation, and the absorbed endotoxin will be rapidly removed by monocytes, particularly resident Kupffer cells within the liver. However, emerging evidence indicates that a chronic, low-level elevation of endotoxin may play a role in insulin-resistant states and obesity [6,7].

Leuwer et al. [8] have shown that endotoxinemia leads to major increases in inflammatory adipokine (TNF-α, IL-6, and MCP-1) gene expression in the white adipose tissue of mice. Indeed, previous studies in human adipose tissue have demonstrated that both states of obesity and T2DM induce an up-regulation of inflammatory genes [7].

Whereas limited data have been presented on the increased levels of endotoxin in pathological conditions such as obesity, diabetes and fatty liver, to date, no studies have compared sedentary and highly trained subjects. Therefore, the objective of the present study was to correlate the plasma levels of endotoxin and inflammatory markers (MCP-1, ICAM/CD54, and VCAM/CD106) between sedentary and highly trained subjects. We hypothesized that individuals that performed large amounts of exercise (moderate/high intensity and long duration) would demonstrate lower levels of endotoxin and inflammatory markers compared to sedentary subjects.

## Methods

### Subjects

Fourteen healthy, non-smoking men participated in this study. The subjects were sedentary (n = 7) [age 28.6 (6.9) years, height 174.0 (0.04) cm, weight 75.6 (10.2) kg] or highly trained athletes in cycling (n = 7) [age 29.8 (5.7) years, height 177.0 (0.06) cm, weight 74.7 (4.4) kg]. The physical and training characteristics of both groups have been described previously by Lira et al. [4]. The benefits and risks were explained before written consent was obtained. The study procedures were previously approved by the Ethics Committee of the Federal University of São Paulo. The non-inclusion criteria were as follows: identified genetic, metabolic or endocrine disease, previous drug utilization, or non-exercise. The athletes avoided exercise for 48 h.

### Blood sampling and analysis

A catheter was inserted in a brachial vein to collect venous blood samples. The blood samples (10 ml) were immediately transferred into two 5-ml vacutainer tubes (Becton Dickinson, BD, Juiz de Fora, MG, Brazil) containing EDTA for plasma separation. The tubes were centrifuged at 3000 × g for 15 minutes at 4°C, and plasma samples were stored at -80°C until analysis. Triglycerides and total cholesterol were assessed using commercial enzymatic kits (Labtest^®^, São Paulo, Brazil). LDL cholesterol was calculated according to Friedewald et al. [11]. PAI-1, MCP1, ICAM/CD54 and VCAM/CD106 were assessed using commercial kits (R&D systems Europe, Ltd. Abingdon Science Park, Abingdon, OX14 3NB, United Kingdom). Some of results obtained for the lipoprotein profiles and characteristics of the subjects have been described previously by our group [4].

### Measurement of circulating endotoxin levels

Plasma endotoxin was assayed using a chromogenic limulus amoebocyte lysate (LAL) test, which is a quantitative test for Gram-negative bacterial endotoxin (Cambrex, Charles City, Iowa, USA). Gram-negative bacterial endotoxin catalyzes the activation of a proenzyme in the LAL. The initial rate of activation is directly determined by the concentration of endotoxin. The activated enzyme catalyzes the splitting of p-nitroaniline (pNA) from the colorless substrate Ac-lle-Glu-Ala-Arg-pNA. The pNA released was measured photometrically at 405-410 nm following termination of the reaction. The correlation between the absorbance and the endotoxin concentration is linear in the range of 0.1-1.0 EU/ml. For the purposes of this study, all samples were run in duplicate within the same plate; therefore, no interassay variability was observed.

To assess the recovery of endotoxin within the assay, known concentrations of recombinant endotoxin (0.25 and 1.00 EU/ml) were added to diluted plasma to determine whether the expected concentration correlated closely with the actual observed value and whether there were any variations due to reactivity with plasma contents. Lyophilized endotoxin (*E. coli *origin) was used to generate a standard curve with the chromogenic LAL test kit from Cambrex according to the manufacturer's instructions.

### Statistical analyses

The distribution of the data was previously checked using Bartlett's test for equal variances, and the data are reported as the means ± standard deviations. The differences in plasma parameters among the groups were assessed using the unpaired t-test. The Pearson correlation coefficient was calculated to assess the relationship between variables. The analysis was carried out using GraphPad Prism (version 5.00) software, and the significance level was set at p < 0.05.

## Results

The subjects demonstrated similar heights, body masses, and body mass indices (Table 1). The weekly exercise intensity, frequency (days), volume (hours) and time experience (years) of the highly trained subjects are also shown in Table 1. A correlation was detected between plasmatic endotoxin and the volume of exercise (positive correlation: lifestyle sedentary, r = 0.55, p < 0.0001, negative correlation: highly trained subjects, r = -0.74, p < 0.001, data not shown).

**Table 1:** Characteristics subjects.

Subjects	Sedentary	Highly Trained Athletes
Height (cm)	174 ± 0.05	177 ± 0.06
Body mass (kg)	75.6 ± 10.2	74.7 ± 4.46
BMI (kg/m^2^)	24.9 ± 2.54	23.9 ± 1.54
Experience exercise training (years)	-	8.86 ± 2.73
Frequence exercise (time for week)	-	4.43 ± 1.27
Volume exercise (hours for day)	-	2.14 ± 0.62
Intensity exercise (VO_2max_)	-	50-100%

Results are expressed as mean value ± SD.

Table 2 show the plasmatic endotoxin, MCP-1, ICAM/CD54, and VCAM/CD106 levels in the two groups. Lower plasmatic levels of endotoxin (p < 0.002) were observed in highly trained subjects compared to the sedentary group. The plasmatic MCP-1 (p < 0.32), ICAM/CD54 (p < 0.43), and VCAM/CD106 (p < 0.17) concentrations were not significantly different between the groups.

**Table 2:** Endotoxin, MCP-1, ICAM/CD54, and VCAM/CD106 levels.

Subjects	Sedentary	Highly Trained Athletes
Endotoxin (EU/mL)	2.05 ± 0.82	0.72 ± 0.28*
MCP-1 (pg/mL)	71.73 ± 21.51	65.03 ± 26.19
ICAM/CD54 (μg/mL)	2.35 ± 0.13	2.37 ± 0.19
VCAM/CD106 (μg/mL)	2.48 ± 0.06	2.53 ± 0.07

Results are expressed as mean value ± SD. *Significantly lower than sedentary group.

**Figure 1:**
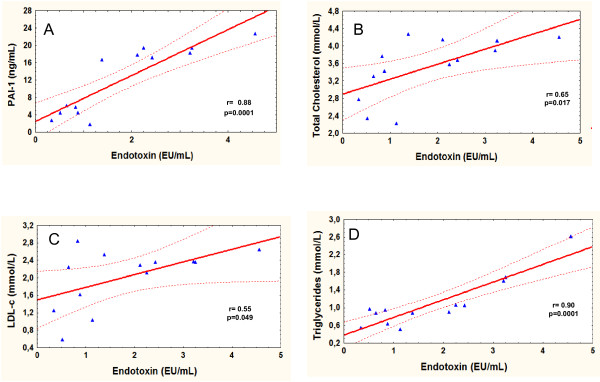
**Correlation between Enddotoxin and PAI-1 (Figure 1a), Endotoxin and total cholesterol (Figure 1b), Enddotoxin and LDL-c (Figure 1c) and Enddotoxin and TG leves (Figure 1d)**.

In addition, positive correlations between endotoxin and PAI-1 (r = 0.88; p < 0.0001 - Figure 1a), endotoxin and total cholesterol (r = 0.65; p < 0.01 - Figure 1b), endotoxin and LDL-c (r = 0.55; p > 0.049 - Figure 1c) and endotoxin and TG levels (r = 0.90; p < 0.0001 - Figure 1d) were observed. No significant correlations were detected between endotoxin and any other variable.

## Discussion

The results of the present study indicate that endotoxin levels may be associated with the lifestyle of the subject. Highly trained subjects that performed large amounts of exercise demonstrated lower levels of plasmatic endotoxin than did the subjects with a sedentary lifestyle. In addition, the plasma PAI-1, total cholesterol, LDL-c and TG concentrations were positively correlated with endotoxin levels. Previous studies have demonstrated that endotoxin levels are elevated in some conditions, such as obesity, diabetes and cardiovascular and fatty liver diseases.

Systemic low-level inflammation has been suggested to be a cause and a consequence of pathological processes associated with the production of local pro-inflammatory molecules functioning as an important biological driver [12]. In the present study, we discovered no differences in the levels of MCP-1, ICAM/CD54 or VCAM/CD106 between sedentary lifestyle and highly trained subjects. Although the subjects with a sedentary lifestyle exhibited higher levels of endotoxin when compared to highly trained subjects, they might have had a low grade of inflammation. Therefore, they might not have presented high levels of MCP-1, ICAM/CD54 or VCAM/CD106, and a progressive, long-term increase in inflammatory factors could lead to the development of obesity, diabetes, and fatty liver. Pedersen [13] described that physical inactivity leads to an accumulation of visceral fat and, consequently, to activation of a network of inflammatory pathways that promote the development of insulin resistance, atherosclerosis, neurodegeneration, and tumor growth, thereby leading to the development of diseases associated with the "diseasome of physical inactivity".

The data presented herein demonstrate for the first time that highly trained subjects show lower endotoxin levels compared to sedentary subjects.

Creely et al. [7] found that circulating serum endotoxin is higher in type 2 diabetes patients than in lean healthy subjects, and endotoxin can activate the innate immune pathway in isolated abdominal adipocytes to stimulate the secretion of pro-inflammatory cytokines. The present authors suggested a role for endotoxin in initiating the subclinical inflammation observed in these conditions. In high-fat diet animal models, Cani et al. [6] observed that metabolic concentrations of plasma endotoxin/LPS are a sufficient molecular mechanism for triggering the high-fat diet-induced metabolic diseases obesity and diabetes. In a recent paper, Harte et al. [10] showed that patients with non-alcoholic fatty liver disease (NAFLD) were characterized by a significant increase in circulating levels of endotoxin. These results were independent of the diabetic status of the patient, and therefore, the authors suggest that endotoxin levels may represent an important early marker of potential liver abnormalities.

Many studies [14-16] have demonstrated the benefits of exercise training, which leads to an anti-inflammatory state in obese rat and human models. Bradley et al. [17] reported that voluntary exercise in diet-induced obese mice reduced adiposity despite continuous consumption of a high-fat diet. In addition, exercise resulted in a normalization of insulin sensitivity and decreased adipose tissue inflammation (reduced IKK-β gene expression) in these animals. These data demonstrated the positive role of exercise training in preventing the development of some diseases, such as obesity, diabetes, and fatty liver.

The protective effect of regular exercise against diseases associated with chronic inflammation may be, to some extent, ascribed to an anti-inflammatory effect that reinforces anti-inflammatory responses. Indeed, it has been suggested and demonstrated by Pedersen's group that cytokines and other peptides are expressed and released by contracting muscle fibers, and they function in a paracrine or endocrine manner and are classified as "myokines" [13]. In a previous study [18], we have shown that adipose tissue also functions as a source of anti-inflammatory factors such as interleukin-10 (IL-10) in chronic exercised rats.

Starkie et al. [19] demonstrated that intravenous endotoxin infusion induces a two to three-fold increase in plasma TNF-α levels. However, when subjects adopted an acute exercise protocol (75% VO2max), the production of TNF-α elicited by low-grade endotoxemia was inhibited in humans. Consistent with the data described above, Chen et al. [20] found that chronically exercised rats exhibited minor pathological changes in the heart, liver, and lung after endotoxemia.

Another factor of interest in the present study was the correlation between the lipid profiles and endotoxin levels. These interrelations may contribute to the development of cardiovascular diseases and fatty liver. Alvarez and Ramos [21] observed that patients hospitalized for various reasons who developed sepsis during their stay showed altered lipoprotein profiles (such as hypertriglyceridemia), and this change was reversed to normal levels following recovery from infections. Using an animal model, Feingold et al. [22] observed that the administration of a high dose of endotoxin (50 μg/100 g body weight) did not increase hepatic fatty acid synthesis or peripheral lipolysis, and furthermore, hepatic triglyceride secretion was not stimulated. Thus, a low dose of endotoxin (10 ng/100 g body weight) produces hypertriglyceridemia by increasing hepatic lipoprotein production, whereas a high dose of endotoxin produces hypertriglyceridemia by decreasing lipoprotein catabolism. In a recent paper, our group demonstrated that sedentary subjects exhibited higher lipoprotein and PAI-1 levels when compared with highly trained subjects [4]. These dysfunctions may contribute to the appearance of diseases.

The limitations of this study include the relatively small numbers of subjects in each of the groups and the lack of subjects with cardiovascular and other diseases.

In summary, our results indicate that sedentary subjects present a favorable state that leads to hypertriglyceridemia and hypercholesterolemia accompanied by high endotoxin levels. In contrast, the performance of highly trained subjects and large amounts of exercise significantly reduced the possibility of risk for cardiovascular diseases.

## Competing interests

The authors declare that they have no competing interests.

## Authors' contributions

FSL, JCR, GDP, HAS, ECC, MCS, LCC, MTM, ARD, LMO and RVTS participed the sample collected, assess samples, design of the study and performed the statistical analysis, and writing of paper. All authors read and approved the final manuscript
